# Organically Grown Food Provides Health Benefits to *Drosophila melanogaster*

**DOI:** 10.1371/journal.pone.0052988

**Published:** 2013-01-09

**Authors:** Ria Chhabra, Santharam Kolli, Johannes H. Bauer

**Affiliations:** 1 Clark High School, Plano, Texas, United States of America; 2 Department of Biological Sciences, Southern Methodist University, Dallas, Texas, United States of America; 3 Center for Drug Discovery, Design and Delivery at Dedman College, Southern Methodist University, Dallas, Texas, United States of America; National Cancer Institute, United States of America

## Abstract

The “organic food” market is the fastest growing food sector, yet it is unclear whether organically raised food is nutritionally superior to conventionally grown food and whether consuming organic food bestows health benefits. In order to evaluate potential health benefits of organic foods, we used the well-characterized fruit fly *Drosophila melanogaster* as a model system. Fruit flies were raised on a diets consisting of extracts of either conventionally or organically raised produce (bananas, potatoes, raisins, soy beans). Flies were then subjected to a variety of tests designed to assess overall fly health. Flies raised on diets made from organically grown produce had greater fertility and longevity. On certain food sources, greater activity and greater stress resistance was additionally observed, suggesting that organic food bestows positive effects on fly health. Our data show that *Drosophila* can be used as a convenient model system to experimentally test potential health effects of dietary components. Using this system, we provide evidence that organically raised food may provide animals with tangible benefits to overall health.

## Introduction

Organic farming aims to preserve soil and ecosystem health by forgoing heavy use of artificial fertilizers and pesticides. In addition to these potential beneficial effects on the environment, consumers are attracted to organic foodstuff because of the claimed positive health effects, presumably due to the absence of pesticides or artificial hormones [1]. Potential detrimental effects of pesticide application may include disruption of neuro-endocrine signaling, negative effects on immune function or the development of cancer, depending on the particular class of pesticide. Especially prenatal exposure, or exposure during infancy, may aggravate these effects (for a review, see [2]). It has been shown that infants consuming a predominantly organic diet have almost non-detectable levels of organo-phosphorus pesticide metabolites [3], suggesting a starting point for investigating molecular mechanisms of the potential health benefits of organic foods.

However, very little is known about the actual health effects of organically farmed food.

Investigations into the nutrient content of organic food have reported increased amounts of vitamins, carotenoids, unsaturated fatty acids and polyphenols [4]–[6]. However, the situation is less clear when the effects of organic food on health are evaluated. Confounding the analysis are issues associated with the transport and storage of foodstuff, the percentage of organic foods in the consumer diet and the kind of organic food consumed [1]. These complications are reflected in a meta-analysis of 50 years worth of data that found no statistically relevant correlation between increased consumption of organic foods and improved health [7].

The absence of a convenient model system to experimentally test the health claims associated with organic foods therefore makes evaluation of any beneficial effect of organically raised foods challenging. In order to develop a better understanding of the potential health benefit of organic food, we used the fruit fly *Drosophila melanogaster* as an easy to use model system. In the past, *Drosophila melanogaster* has been successfully used to investigate biological problems such as genetics or developmental biology [8]. Over the last decade, *Drosophila* has increasingly been used to model human conditions such as immune [9] and cardiac function [10], as well as neurodegenerative diseases [11], infectious diseases [12] and aging [13]. In addition, *Drosophila* models for metabolic disease have recently been developed: In a model of sucrose overfeeding, *Drosophila* larvae show symptoms consistent with Type 2 diabetes [14]. Moreover, when the insulin-producing cells (IPC) are ablated, adult *Drosophila* show signs of Type 1 diabetes. Interestingly, when these IPC-ablated flies are injected with insulin, those symptoms are alleviated [15].

It is well known that dietary factors affect *Drosophila* fertility, longevity and health. High fat diets have been shown to lead to cardiac dysfunction [16]. Very low and high caloric diets shorten fly life span, presumably due to the unhealthy effects of under- and overfeeding, respectively. Longest life spans are usually observed at low calories, short of underfeeding, a situation referred to as Calorie Restriction [17]. Interestingly, dietary carbohydrates and protein have largely opposing effects on fly physiology, with high carbohydrate loads leading to increased weight gain and fat accumulation and high protein diets leading to highly fertile and leaner flies [18]. Our own work on diet-dependent disruption of metabolic homeostasis demonstrates that overfed adult flies develop metabolic abnormalities, dependent on which macronutrient was fed in excess. Flies fed a carbohydrate rich diet shows signs of Type 2 diabetes, while flies on high protein diets show signs of ketosis. Under both conditions, flies develop insulin-resistance, a hallmark of Type 2 diabetes [19]. These data suggest that the fly model can be successfully used to investigate issues concerning nutrition, mammalian metabolism and health.

Here, we report on the use of the *Drosophila* model to assess the health benefits of organically farmed produce. Flies were raised on a variety of diets and their overall health evaluated. Flies raised on organic food showed improved performance on most tests, such as increased fertility and stress resistance.

## Materials and Methods

### Fly culture and diet preparation

The wild type strain Canton-S was obtained from the Bloomington *Drosophila* Stockcenter at Indiana University (Bloomington, IN). All flies were kept in a humidified (50%), temperature-controlled incubator with 12 hour on/off light cycle at 25°C in vials containing standard cornmeal medium [20].

Experimental, produce-based diets were prepared by homogenizing 1500 g of the indicated items in a Hamilton blender. Homogenized produce was then mixed in 1 L ddH_2_O (final volume) containing 10% agarose. After autoclaving, tegosept to a final concentration of 2.3 g/l was added and 5 ml of food was dispensed into individual vials.

Produce was purchased from a local outlet of the Whole Foods national chain of supermarkets (Whole Food Markets, Austin, TX) that carries both organic and conventional produce. Whole Foods is a leading national retailer of organic food items in the US and was instrumental in establishing a national organic food marketplace and setting national standards for the certification of organic foods. Organic produce is manufactured according to USDA guidelines outlined in the National Organic Program (NOP, http://www.ams.usda.gov/AMSv1.0/nop).

Estimation of the nutritional content of the selected food items was based on USDA guidelines (http://www.ars.usda.gov/Services/docs.htm?docid=6282).

### Life span analysis

Newly eclosed flies were collected under light anesthesia, randomly divided into treatment groups and housed at a density of 25 males and 25 females each per vial. At least ten such vials were used per treatment as per [21]. Flies were passed every other day and the number of dead flies was recorded.

### Stress resistance

For assays testing resistance to starvation and H_2_O_2_ (SIGMA), at least 8 vials of newly eclosed flies containing 25 males and females each were collected and aged for ten days under the same conditions as for life span analysis. For starvation assays, flies were then shifted to vials containing a 2% agar matrix to avoid desiccation. For H_2_O_2_ assays, flies were shifted to vials containing 2% agar with 5% sucrose/5% H_2_O_2_. The number of dead flies was counted twice daily.

### Physical activity measurements

For activity measurements, flies were collected and cultured as for life span assays. At 10 days, flies were separated by sex, and measurements were taken in a LAM25H-3 Locomotor Activity Monitor (Trikinetics Inc.) over at least a 48 hr period using 20 animals per vial. Activity was recorded every 10 minutes.

### Fertility

Fertility was examined using vials containing 10 males and 10 females each. Flies were passed and eggs were counted daily over a 10-day period.

### Quantitative PCR

Total mRNA was isolated from at least 75 10-day old females using Trizol (Invitrogen) and further purified using the RNeasy kit (Qiagen). cDNA was generated with 0.5 µg total mRNA in a 10 µl reaction using the iScript cDNA synthesis kit (BioRAD). 0.8 µl of the iScript reaction was used as QPCR template. QPCR was performed as described [22] on a BioRAD CFX96 RealTime PCR System using the ABI SYBR-Green PCR master mix following the manufacturers instructions. Each QPCR reaction was performed using at least two biological replicates in triplicate each.

### Statistical analysis

Log-rank tests for survivorship curves and two-way ANOVA (fertility) and t-tests (QPCR) were performed using the Prism suit of biostatistical software (GraphPad, San Diego).

## Results and Discussion

In order to test directly whether organically farmed food elicits beneficial health effects, we raised fruit flies on diets made from extracts of different produce without any additional supplementation. We then subjected the flies to a series of experiments to determine their overall health.

**Figure 1:**
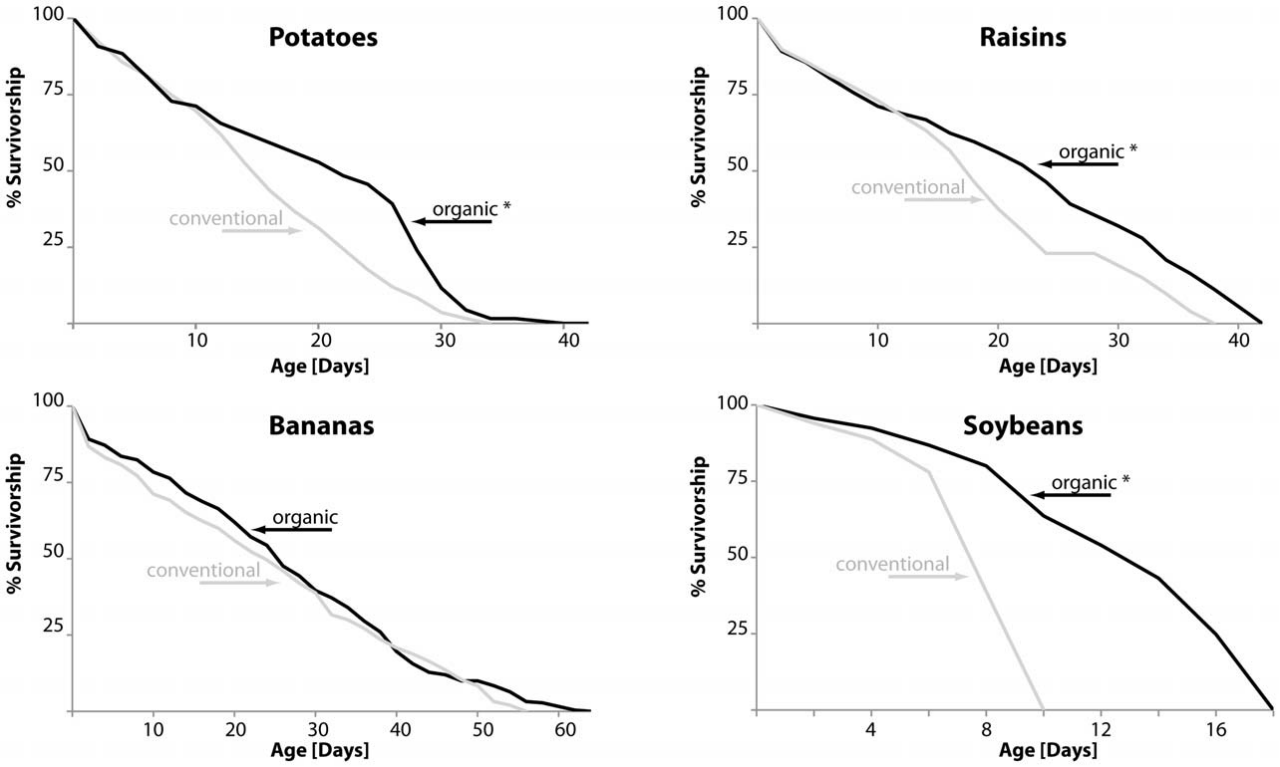
Longevity of D. *melanogaster* fed organic diets. Survivorship curves of female fruit flies fed diets made from extracts of potatoes, raisins, bananas or soybeans (grey: conventional food; black: organic food; statistically significant changes (p<0.005) are indicated by asterisks). Median survival times of flies on conventional and organics food sources, respectively, are: potatoes: 16 and 22 days (∼38% longevity increase, p<0.0001); raisins: 2 and 24 days (∼20% longevity increase, p<0.0001); bananas: 24 and 26 days (p = 0.1543); soybeans: 8 and 14 days (∼75% longevity increase, p<0.0001).

Longevity and fertility are the most important life history traits of an animal and are excellent indicators for overall health. *Drosophila* cultured on produce extract diets were generally shorter lived than flies raised on regular lab food, presumably due to limited nutritional balance in diets prepared from a single produce source. This is represented in the survivorship curves, which do not possess the usual sigmoidal shape, but rather resemble relatively straight lines (Figure 1). Nonetheless, flies raised on organic potato, raisin or soy diets had significantly extended longevity compared to flies raised on conventional produce extracts, while flies raised on an organic banana diet had similar longevity to flies raised on conventional banana food (Figure 1, for full statistical analysis please refer to Table S1).

**Figure 2:**
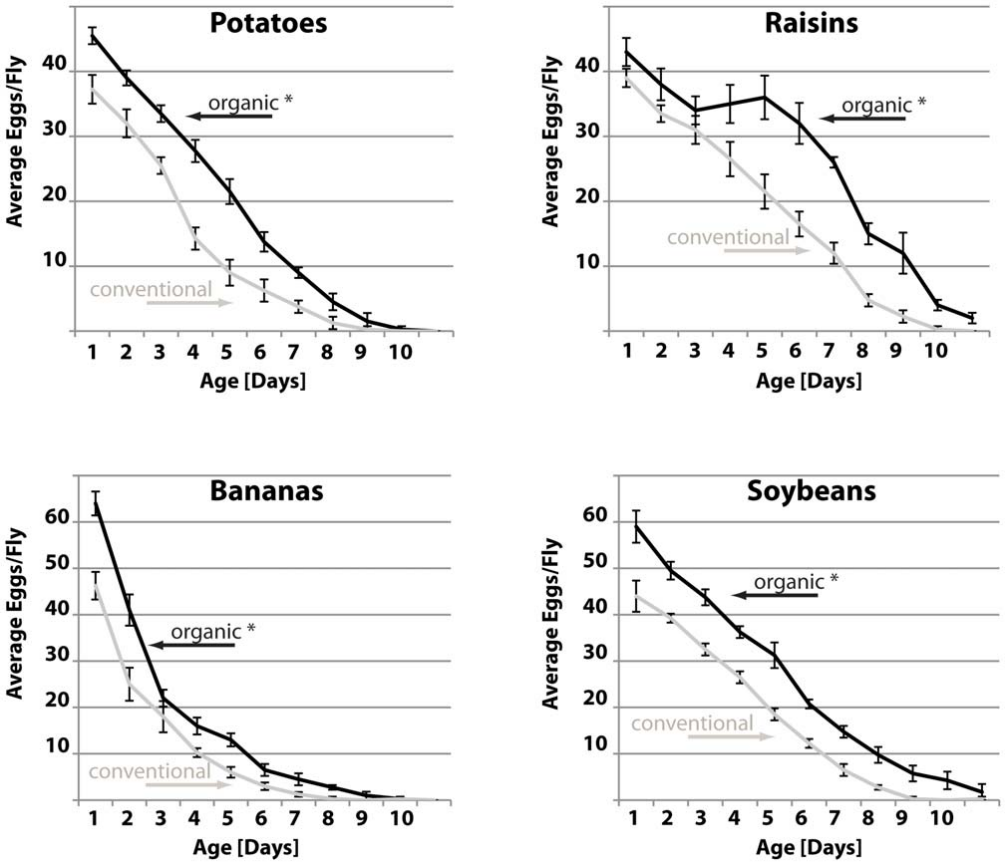
Daily egg-laying of flies exposed to organic diets. Egg production of flies fed the indicated food was determined daily. Shown are the averages of four biological replicates; error bars represent the standard deviation (grey: conventional food; black: organic food; statistically significant changes (p<0.005) are indicated by asterisks; p<0.0001 for all food types).

Next, we tested the fertility of flies raised on the organic diets versus flies raised on conventional diets. As shown in Figure 2, flies fed extracts of any organic produce had significantly higher daily egg production than flies fed conventional diets. Interestingly, flies fed the normal balanced laboratory diet have significantly higher fertility, with an egg production peak between five and ten days, while flies fed the produce extract had steadily declining fertility levels, reminiscent of what is observed with longevity. Due to the extremely short life spans of flies raised on soy diets, flies raised on soy diets were excluded from all subsequent assays.

**Figure 3:**
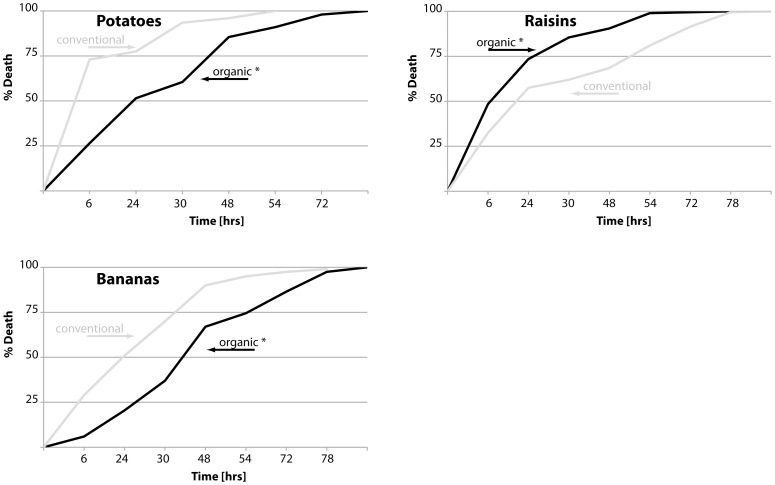
Starvation tolerance of flies raised on organic diets. Survivorship curves of female flies raised for 10 days on the indicated food sources. Flies were then transferred to starvation media and dead flies were counted twice daily (grey: conventional food; black: organic food; statistically significant changes (p<0.005) are indicated by asterisks). Median survival times of flies on conventional and organics food sources, respectively, are: potatoes: 6 and 24 hours (p<0.0001); raisins: 24 and 24 hours (p<0.0001); bananas: 24 and 48 hours (p<0.0001).

These data suggest that organic foods are more nutritionally balanced than conventional foods, or contain higher levels of nutrients, leading to improved fertility and longevity. In order to further investigate whether organic food provided nutritional benefits, we determined the survival times of flies when starved. Flies raised on organic potato extracts survived wet starvation significantly longer than flies fed conventional potato extract, suggesting a higher nutritional value of the organic extract. No change in survival time was observed with organic banana extract, while flies fed organic raisin extract had decreased survival times (Figure 3, for full statistical analysis please refer to Table S1).

**Figure 4:**
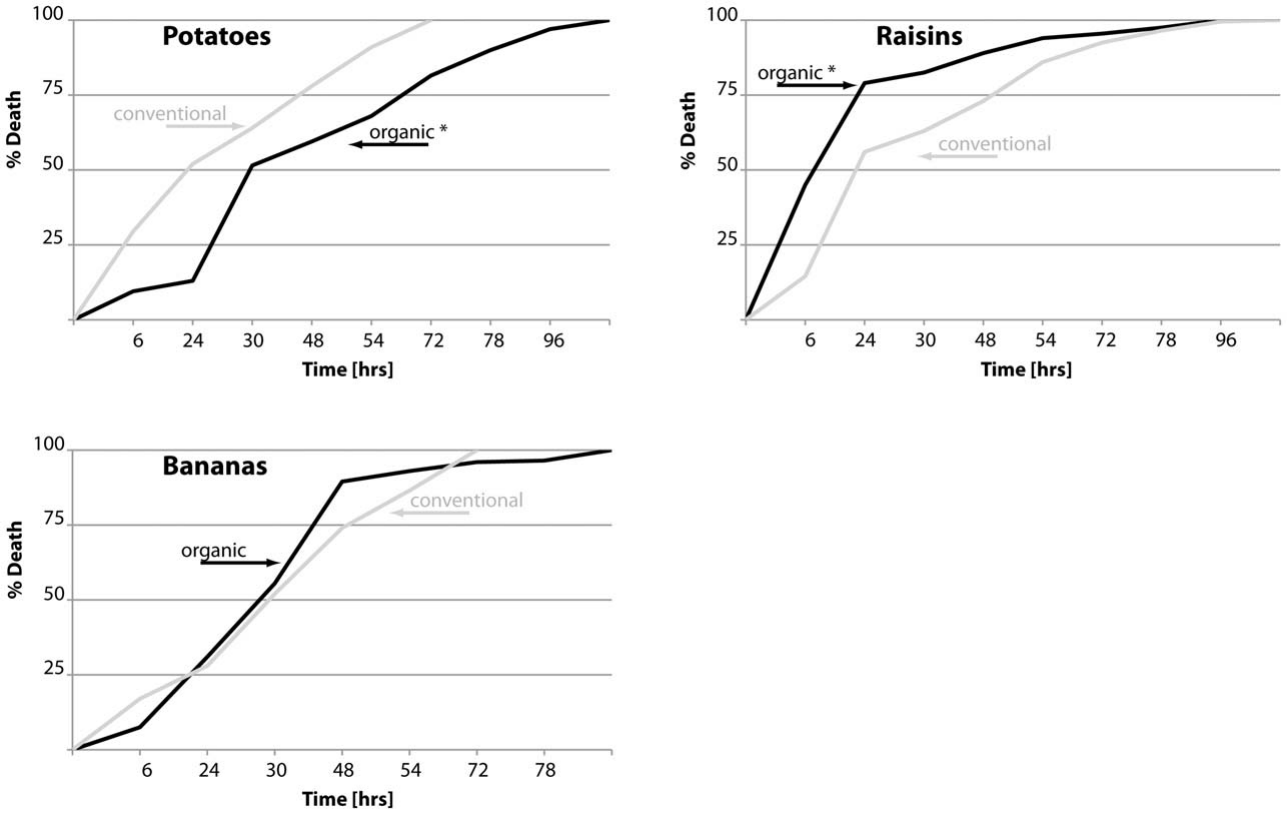
Oxidative stress resistance of *Drosophila* raised on organic food. Survivorship curves of female flies raised for 10 days on the indicated food sources. Flies were then transferred to media containing H_2_O_2_ and dead flies were counted twice daily (grey: conventional food; black: organic food; statistically significant changes (p<0.005) are indicated by asterisks). Median survival times of flies on conventional and organics food sources, respectively, are: potatoes: 24 and 30 hours (p<0.0001); raisins: 24 and 24 hours (p<0.0001); bananas: 30 and 30 hours (p<0.2172).

We next measured the oxidative stress resistance of flies fed the organic diets in order to test whether organic food provided protection against oxidative damage. As shown in Figure 4, flies raised on organic potato or banana diets, but not raisin diets, survived this treatment longer than conventionally fed flies (for full statistical analysis please refer to Table S1). In addition, we measured the spontaneous activity of flies fed organic diets over a 48-hr period. As shown in Figure S1, flies raised on extracts of organic raisin and banana food had higher overall activity than flies fed the conventional diets.

The organic food market is rapidly growing, partly due to the consumers' perception of superior nutritional quality of organic foodstuff. However, verification of any health claims associated with organic food is complicated by the lack of an adequate system to experimentally test those claims [1]. Several groups attempted meta-analyses of the available reports in the literature to assess the qualities attributed to organic food items, but this large-scale review of the literature provides conflicting results. In one such study, the authors find that organic foods generally have higher amounts of vitamins and polyphenols, as well as higher amounts of essential amino acids [23]. In contrast, a similar very recent analysis of ∼50 years worth of data concluded that the hypothesis that organic food has higher nutritional value is not supported [24]. Nonetheless, both studies found that organic food had significantly lower levels of pesticide contamination. While this finding may support the hypothesis that organic food has health benefits, no evidence for this has been presented so far [4], [7], [24].

We addressed this unsatisfactory situation by performing preliminary analysis using a simple and convenient model system, the fruit fly *Drosophila melanogaster*. Recent advances have demonstrated that *D. melanogaster* can be successfully used to model the consequences of disrupted metabolic homeostasis on fly health parameters [14], [15], [19]. Advantages of the fly system lie in its cheap operating costs and its short generation time, which allows for rapid testing of multiple interventions. We therefore used the fly model to investigate whether certified organic produce provided any health benefits to fruit flies.

In order to determine whether organically raised food provided health benefits, we raised fruit flies on a variety of diets made from produce extracts. Using this regime, we were able to test the effects of each food type independently, thus avoiding confounding effects of a mixed diet. Interestingly, flies raised on produce extracts had shorter life spans and reduced fertility compared to flies raised on regular lab food (data not shown). These data suggest that single-component produce extracts do not provide a nutritionally balanced diet for fruit flies. However, this observation provided a convenient platform to determine the health effects of organic foods. We performed tests measuring the longevity, fertility and stress and starvation resistance of flies raised on organic food extracts versus flies raised on conventional food extracts. Our data demonstrate that flies raised on organic food extracts by and large performed better on the majority of health tests (summarized in Table 1). *Drosophila* raised on diets based on organic foods performed better on 13 of 17 independent tests (15 of 19 if the activity data is considered). Interestingly, almost all negative or neutral results were obtained using raisin diets, suggesting the beneficial health effects of organic diets are dependent on the specific food item, which may explain some of the inconsistent results in the literature. Most importantly, our organic fed fruit flies showed improvements on the most significant measures of health [25]: fertility and longevity (flies raised on organic banana food had an 8% longevity increase that was not quite statistically significant). These data thus demonstrate that the fly system can successfully be used to evaluate health benefits of individual food items, thus providing a convenient tool for nutritional studies.

**Table 1:** Effects of organic diets on *Drosophila* health parameters compared to conventional diets.

Diet	Longevity	Fertility	Starvation	Ox. Stress	Activity	QPCR: DILP/gluconeogenesis
Potato	↑	↑	↑	↑	ND	↑
Raisin	↑	↑	↓	↓	↑	NC
Banana	NC	↑	↑	NC	↑	↑
Soybean	↑	↑	ND	ND	ND	ND
↑: increased	↓: decreased	NC: no change	ND: not determined			

Initially, we only tested banana extract food, due to the historic use of banana extract as a food source when *Drosophila* was first cultivated as a laboratory animal by the group of T. H. Morgan. Since then, standard *Drosophila* laboratory food has been designed that contains sucrose as a carbohydrates source and yeast extract as a protein source. As bananas are carbohydrate-rich and protein poor, we decided to include other produce sources with different nutritional profiles (for example soy beans as a high protein content food source). Expanding the range of produce tested therefore addresses the question of macronutrient-specificity of the observed effects.

An additional concern is that the organic and conventional produce was not matched for soil condition, latitude of growth etc. However, this situation resembles what a consumer might encounter in the store, where organic and conventional produce items from different growing regions exist side-by-side. We therefore tested a 100% organic vs. 100% conventional diet in our fly model system with non-matched produce sources. Nonetheless, irrespective of the predominant macronutrient or individual growth condition, organic food by-and-large provided positive effects under our experimental conditions, suggesting that the organic production process may provide benefits to the consumer.

**Figure 5:**
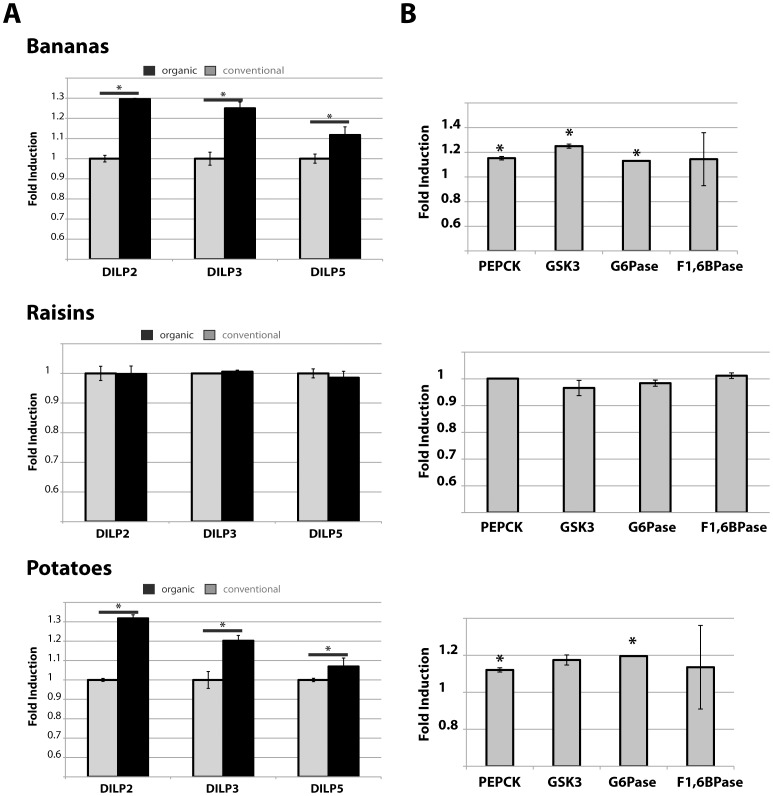
QPCR analysis of fruit flies raised on organic diets. Flies were raised for ten days on the indicated diets and mRNA was isolated from whole body extracts of females. Levels are shown as fold-induction compared to the mRNA levels of flies raised on conventional diets (A) Levels of the indicated DILP mRNAs were normalized against beta-tubulin (shown are the averages of five biological replicates; error bars represent the standard deviation; statistically significant changes (p<0.005) are indicated by asterisks; grey: conventional food; black: organic food). (B) mRNA levels of the indicated metabolic genes were normalized against rp49 (shown are the averages of two experiments; error bars represent the standard deviation; statistically significant changes (p<0.05) are indicated by asterisks).

Our data suggest that organic foods provide improved health outcomes. The reason for this effect remains unclear. In an attempt to investigate the molecular mechanism for these improved health effects, we measured the levels of insulin in flies raised on organic foods. We investigated the mRNA levels of three of the seven *Drosophila* insulin-like peptides (DILP) [26]. These three DILPs are secreted from median secretory neurons, the insulin-producing cells, which are functionally similar to mammalian pancreatic β-cells [26]. The specific roles of these DILPs in regulating fly physiology are still unclear, as knockdown of each individual DILP does not lead to major abnormalities [27], suggesting redundancy between individual DILPs. Nonetheless, DILP2 has been suggested to be important for longevity regulation [21], [28], [29], while DILPs3 and 5 may be important for the regulation of developmental timing and fly growth [27]. Compared to flies raised on conventional food, mRNA levels of these DILPs were slightly increased in flies raised on organic potato and banana, but not raisin, extract (Figure 5A). Increased insulin-levels are often observed in situations of insufficient insulin-signaling activity, such as hyperinsulinemia in insulin-resistance, which can be observed in fruit flies [19]. Interestingly, down regulation of insulin-signaling has been shown to increase health and life span of *D*. *melanogaster* [30], *C. elegans* [31] and rodents [32]. We therefore tested whether organic fed flies have lower levels of insulin-signaling activity by investigating the mRNA levels of several genes involved in gluconeogenesis, such as glycogen-synthase 3 (GSK3), phosphoenolpyruvate carboxykinase (PEPCK), glucose-6-phosphatase (G6Pase) and fructose-1,6-bisphosphatase (F1,6BPase). As shown in figure 5B, flies raised on organic raisin food had no changes in the levels of these four genes, while flies raised on either organic banana or potato diets showed weak upregulation of mRNA levels. Despite significant p-values, these changes in mRNA levels are close to the detection limit of QPCR and thus may be too small to be considered significant. Therefore, these data suggest that reduced insulin-signaling does not play a role in mediating health effects of organic produce.

Several studies have shown that organic food contains higher levels of essential nutrients, such as an increase in total protein content and unsaturated fatty acids in dairy products [4], or an increase in antioxidants in spinach [33], tomatoes [6] or bell peppers [34]. Organic food has furthermore been shown to contain lower levels of nitrates [33], which may explain some of the improved health characteristics of *Drosophila* raised on organic foods. Interestingly, organic foods have been demonstrated to contain elevated levels of polyphenols [4]–[6]. Polyphenols are organic compounds produced by many plants to fight diseases. However, polyphenols have beneficial health effects on animals, possibly through a mechanism such as xenohormesis [35]. The xenohormesis hypothesis postulates that the stress-related accumulation of certain plant molecules, such as polyphenols, may be sufficient to elicit a hormetic response in animals consuming those plants. Therefore, decreased pesticide and fungizide applications in organic farming may induce plants to upregulate production of their own defense and stress systems, which in turn could elicit beneficial xenohormetic responses upon consumption by animals.

The exact molecular mechanisms of the observed health effects remain to be elucidated. Altered insulin-signaling, altered redox balance or xenohormesis may all play a role. The use of the *Drosophila* model will be invaluable not only in investigating potential health effects of a variety of food sources, but furthermore in dissecting the molecular pathways underlying the health effects of organic foods.

## Supporting Information

Figure S1 **Spontaneous activity of fruit flies.** The spontaneous activity and circadian rhythm of female fruit flies was measured after aging to 10 days on the indicated diets. No difference in circadian patterns or activity during regular resting phases was observed, but flies raised on organic raisin or banana food displayed higher activity during regular periods of activity (grey: conventional food; black: organic food).
(TIF)

Table S1 Dietary effects on female survivorship.
(DOC)
