# Supplementary figures and images for: Organically Grown Food Provides Health Benefits to Drosophila melanogaster
Source: PLoS One. 2013 Jan 9;8(1):e52988. doi: 10.1371/journal.pone.0052988 (PMC3541339; doi:10.1371/journal.pone.0052988)

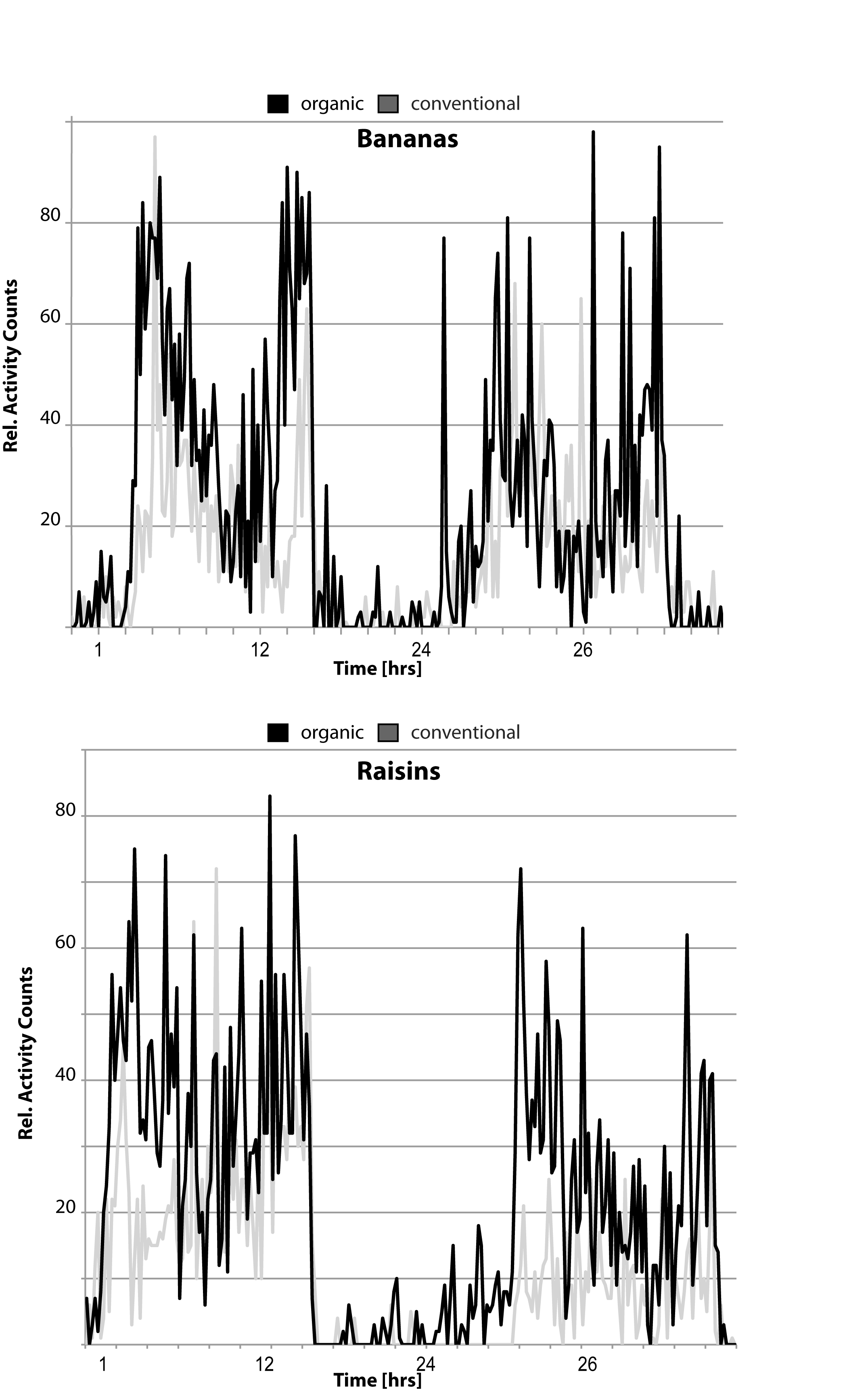

Supplement: Figure S1 — Spontaneous activity of fruit flies. The spontaneous activity and circadian rhythm of female fruit flies was measured after aging to 10 days on the indicated diets. No difference in circadian patterns or activity during regular resting phases was observed, but flies raised on organic raisin or banana food displayed higher activity during regular periods of activity (grey: conventional food; black: organic food). (TIF) [file pone.0052988.s001.tif]
