# Supplementary material for: Organically Grown Food Provides Health Benefits to Drosophila melanogaster
Source: PLoS One. 2013 Jan 9;8(1):e52988. doi: 10.1371/journal.pone.0052988 (PMC3541339; doi:10.1371/journal.pone.0052988)
Supplement: Table S1 — Dietary effects on female survivorship. (DOC) [file pone.0052988.s002.doc]

# Supplemental Table 1

Dietary effects on female survivorship.

|  | **Median LS (organic vs. conventional)** | **Median LS**  **extension** | **Max LS (organic vs. conventional)** | **Max LS**  **extension** | **Number of flies**  **(conventional;**  **organic)** | **χ2** | **p-value** |
| --- | --- | --- | --- | --- | --- | --- | --- |
| **Longevity (days)** |  | | | | | | |
| bananas | 26/24 | 8.3% | 56/52 | 7.7% | 250  250 | 2.029 | 0.1543 |
| raisins | 24/20 | 20% | 41/36 | 13.9% | 250  250 | 21.48 | <0.0001 |
| potatoes | 22/16 | 37.5% | 32/30 | 6.7% | 250  250 | 33.27 | <0.0001 |
| soy beans | 14/8 | 75% | 18/10 | 80% | 250  250 | 189.9 | <0.0001 |
| **Starvation (hrs)** |  | | | | | | |
| bananas | 48/24 | 200% | 78/63 | 23.8% | 200  200 | 58.73 | <0.0001 |
| raisins | 24/24 | 0% | 54/78 | -30.8% | 200  200 | 38.59 | <0.0001 |
| potatoes | 24/6 | 400% | 72/54 | 33.3% | 200  200 | 72.53 | <0.0001 |
| **Peroxide (hrs)** |  | | | | | | |
| bananas | 30/30 | 0% | 72/72 | 0% | 200  200 | 1.523 | 0.2172 |
| raisins | 24/24 | 0% | 69/78 | -11.5% | 200  200 | 40.03 | <0.0001 |
| potatoes | 30/24 | 25% | 96/72 | 33.3% | 200  200 | 58.07 | <0.0001 |

**Table 1:** Log rank analysis of the survivorship curves of female flies. Mean, median and maximum lifespan, log rank analysis, p-value, percent change in mean, median and maximum lifespan as compared to controls (without RU486 for GeneSwitch experiments), Chi-square and p-values derived from the survivorship curves for each indicated intervention are shown. Maximum life span was calculated as the median life span of the longest surviving 10% of the population.
